# Virtual pulmonary rehabilitation approaches in patients with post COVID syndrome: a pilot study

**DOI:** 10.1186/s12890-024-02965-3

**Published:** 2024-03-18

**Authors:** Antonio Sarmento, Rachel Adodo, Greg Hodges, Sandra C. Webber, Diana C. Sanchez-Ramirez

**Affiliations:** 1 Department of Respiratory Therapy, University of Manitoba, Room 334 - 771 McDermot Ave, Winnipeg, MB R3E 0T6 Canada; 2 Health Sciences Centre. Winnipeg, Winnipeg, MB Canada; 3 Department of Physical Therapy, University of Manitoba, Winnipeg, MB Canada

**Keywords:** Post-COVID syndrome, Virtual, Telerehabilitation, Pulmonary rehabilitation

## Abstract

**Background:**

The characteristics of optimal virtual pulmonary rehabilitation (PR) for individuals with post-COVID syndrome (PCS) have not been identified. This study aimed to assess the feasibility, safety, and satisfaction associated with a virtual PR program with the exercise component delivered through group or self-directed sessions.

**Methods:**

Adults with PCS-respiratory symptoms were randomly assigned to the video conference (PR_VC_) or self-directed (PR_SD_) group and completed an exercise program (aerobic, strengthening, and breathing exercises) three times/week for eight weeks. PR_VC_ sessions were led by a physiotherapist via Zoom, whereas the PR_SD_ group exercised individually following a pre-recorded video. Both groups received personalized exercise recommendations, education related to the condition, and a weekly follow up call. Satisfaction was assessed through a patient survey. Lung function, dyspnea, fatigue, sit-to-stand capacity, health-related quality of life, and participation were assessed pre- and post-PR.

**Results:**

Fourteen PCS individuals (49 ± 9 years, 86% females) completed 83% of the sessions. All participants were satisfied with information provided by the therapist and frequency of data submission, whereas most were satisfied with the frequency and duration of exercise sessions (88% in PR_VC_ and 83% in PR_SD_). A higher proportion of participants in the PR_VC_ (88%) were satisfied with the level of difficulty of exercises compared with the PR_SD_ (67%), and 84% of the sample reported a positive impact of the program on their health. No adverse events were reported. Significant changes in sit-to-stand capacity (*p* = 0.012, Cohen’s *r* = 0.67) and questions related to fatigue (*p* = 0.027, Cohen’s *r* = 0.58), neurocognitive (*p* = 0.045, Cohen’s *r* = 0.53), and autonomic (*p* = 0.024, Cohen’s *r* = 0.60) domains of the DePaul Symptom Questionnaire short-form were also found between groups.

**Conclusion:**

Virtual PR with exercises delivered via video conference or pre-recorded video were feasible, safe, and well-received by individuals with PCS.

**Trial registration:**

NCT05003271 (first posted: 12/08/2021).

**Supplementary Information:**

The online version contains supplementary material available at 10.1186/s12890-024-02965-3.

## Background

Apart from the severe morbidity and high mortality caused by the coronavirus disease 2019 (COVID-19), approximately 10%–20% of people worldwide experience the continuation or development of new symptoms for more than three months after the initial SARS-CoV-2 infection [1]. These symptoms are characteristic of post COVID syndrome (PCS), a complex multisystem condition that may last years and severely impair lung function, exercise capacity, activities of daily living, and quality of life [2–4]. PCS can also affect the ability of patients to return to work, causing an economic burden for the individual, family, and society [5, 6]. Therefore, appropriate interventions must be identified to support the recovery of individuals with this condition.

Pulmonary rehabilitation (PR) has been shown to improve dyspnea, fatigue, exercise capacity, health-related quality of life (HRQoL), and physical function in people with chronic respiratory diseases [7–9]. Despite these benefits, participation in PR programs is still limited, mainly due to lack of access to programs, distance to centers, and mobility restrictions [10–12]. In this context, home-based PR programs may provide an easier, practical, less-costly, and effective alternative to in- and outpatient programs [13, 14]. They were rapidly adopted during the COVID-19 pandemic to overcome many mobility restrictions, facilitate access, and reduce the healthcare system burden [15–17].

Recent advances in technology development, including communication platforms and portable devices, have facilitated social interaction and the delivery of virtual programs [18, 19]. Wearable devices have also helped monitor patients to safely engage in remote exercises [20, 21]. Evidence suggests that virtual rehabilitation positively affects outcomes of individuals with chronic conditions and may be as effective as standard care [22]. In this sense, virtual PR may be a viable alternative for this population to overcome the barriers to accessing rehabilitation services and for healthcare providers to support the long-term management of individuals with PCS. However, the characteristics of optimal virtual interventions for these individuals have not been identified. Therefore, this study aimed to (1) assess the feasibility, safety, and satisfaction of a virtual PR program in which exercises were delivered via group or self-directed sessions, and (2) explore its effects on lung function, dyspnea, fatigue, sit-to-stand capacity, HRQoL, and participation of individuals with PCS-related respiratory symptoms.

## Methods

### Ethics, recruitment, and eligibility criteria

This pilot study used a two-arm randomized pre- and post-trial design. The study was registered in the ClinicalTrials.gov platform (NCT05003271–12/08/2021) and approved by the research ethics committee of the University of Manitoba (number HS251-80 B202:101). All participants signed the informed consent form.

A convenience sample of 21 adults aged ≥ 18 years, complaining of mild to severe persistent respiratory symptoms ≥ 3 months after confirmed or suspected COVID-19 infection, with home internet, and access to a smart device (phone, tablet, or computer) were recruited via public advertising (local radio and TV) or social media. Exclusion criteria were history of neurological or mental diseases; inability to ambulate independently without supervision; and inability to complete basic tasks on a smart phone or tablet, such as searching, opening, and closing an app. Those who did not return calls after the initial contact or declined to participate before randomization were also excluded.

### Procedures

Participants who contacted the research team expressing interest in the study were screened by phone. Those who met the inclusion criteria signed the informed consent and were randomly assigned to one of the following two groups using the website randomlists.com (1:1 block randomization): video conference (PR_VC_) or self-directed (PR_SD_) exercises. Participants received an e-mail with information about the virtual PR according to their assigned group. An envelope was also mailed to the home of participants with a printed version of the questionnaires, exercise program, and activity diary; one portable spirometer (SpiroBank Smart, MIR, Rome, Italy), nose clip, and three disposable mouthpieces with turbines; one digital finger pulse oximeter (LOOKEE®, New York, USA); and one prepaid envelop for returning the equipment and the diary after the study. Once the participant received the envelope, an individual appointment was scheduled via video conference to explain study procedures, questionnaires, and equipment use; collect demographic data; conduct the initial assessment; and provide education and personalized recommendations for exercise.

### Study protocol

#### Pulmonary rehabilitation program

After an initial assessment, all individuals took part in an eight-week virtual PR program in which the same exercise components were delivered via group sessions (PR_VC_) or self-directed (PR_SD_).

Participants in the PR_VC_ were asked to join a live 30-minute exercise program with a small group of peers (6 participants each) via video conference three times a week. The PR_VC_ exercise program was comprised of three phases (warm-up, resistance and aerobic exercises, and cool down) (Additional file 1) and time (5 min before and 10 after the exercise program) to ask questions, share information, or have an informal interaction; thus, the total session time was 45 min. All sessions were led by a physiotherapist, who also resolved general questions about exercises, equipment, or the video conference platform. Participants in the PR_SD_ were asked to perform the same exercise program at home three times a week following a pre-recorded video created by the research team and uploaded on YouTube; the exercises performed by the PR_SD_ were unsupervised.

Personalized recommendations regarding maximum heart rate (HR) and minimum oxygen saturation (SpO_2_) during exercise [23, 24], and instructions on safety precautions (i.e., when to seek professional or emergency care) were given to all participants. They also received instructions on how to (1) use the modified Borg Scale (exercise intensity should be between 4 and 6), (2) use the pulse oximeter during exercises (self-monitor) to control the pace and avoid exceeding the target HR and SpO_2_, and (2) record HR and SpO_2_ in a diary before and after each exercise session. Exercises or activities performed by participants between the three weekly sessions were not controlled.

Participants received education related to their condition (e.g., pacing strategies and managing breathlessness, activities of daily living, stress, and problems with attention, memory, and thinking clearly) [25] and were trained in the basic management of video conferences (e.g., joining and leaving a Zoom meeting) or Youtube platform depending on the assigned group, the use of the portable spirometer, and the process to send lung function, HR, and SpO_2_ results to the team (e-mail or SMS) once per week. They were encouraged to contact the physiotherapist by e-mail or phone at any time during the study in case of questions or concerns. All participants received a phone call once a week to answer questions and follow up on the general symptoms. When necessary, exercises were adapted by the physiotherapist (e.g., increase the number of repetitions or resistance) according to the symptoms and perceptions of participants. For the PR_VC_, modifications were made during the video conference, whereas adjustments for the PR_SD_ were made during the weekly call.

### Assessments

For both groups, the initial assessment was conducted during a video conference by a physiotherapist, who collected data about sex (female, male, or other), age, self-reported height (cm) and weight (kg), smoking history, comorbidities, time since infection (< 3 months, between 3 and 6 months, or > 6 months), COVID-19 severity (mild, severe, or critical according to the main setting in which individuals received treatment [i.e., home, hospital, or ICU, respectively]), self-reported physical activity level before COVID-19 (sedentary, mild, moderate, or high) [26], and use of respiratory equipment (e.g., non-invasive ventilation) during and after COVID-19 (yes or no). Secondary outcomes were collected at the initial and final assessments.

### Primary outcome

The primary outcome measures were the feasibility and its indicators (recruitment rate, intervention completion rate, and dropout rate), safety, and satisfaction with the proposed PR program.

#### Feasibility

The feasibility for implementing a virtual PR program incorporating exercise approaches was determined according to the following criteria: (1) 70% of participants completed the PR program, (2) data on primary outcomes collected in ≥ 70% of participants after the PR program, and (3) < 10% of adverse events related to the intervention [27].

#### Recruitment rate

The percentage of potentially eligible participants that were recruited was considered the recruitment rate.

#### Intervention completion rate

Completion rate was represented as the proportion of sessions attended/completed by participants [28, 29]. The number of sessions in the PR_VC_ was recorded by the physiotherapist who attended video conferences, whereas those in the PR_SD_ were asked to record the sessions in the diary.

#### Dropout rate

Dropout rate [30] was defined as the proportion of individuals who ceased participation after randomization and before completing 80% of sessions due to adverse events or personal preferences [31].

#### Safety

Safety was considered as the proportion of breathing and fatigue symptoms pre- and post-virtual PR and the incidence of adverse events caused by the interventions (e.g., exacerbation of the condition, musculoskeletal injuries, pain, medical emergencies, falls, and severe dyspnea) [32].

#### Satisfaction with the program

Satisfaction was evaluated during the final assessment using a questionnaire developed by the team, which included questions about the program (information provided, duration and frequency of sessions, level of difficulty of exercises, impact on overall health, and overall satisfaction), data collection (duration and frequency), apps (installation and use), devices (use and technical difficulties), and support received. Answers were provided using a scale from 1 (strongly disagree) to 5 (strongly agree). Suggestions and comments were also collected.

### Secondary outcomes

#### Lung function

Forced vital capacity (FVC), forced expiratory volume in the first second (FEV_1_), FEV_1_/FVC, and peak expiratory flow were assessed using a SpiroBank Smart spirometer (MIR, Rome, Italy) and the associated MIR SpiroBank app, according to ATS/ERS recommendations [33]. Data were compared to reference values for the Canadian population [34].

#### Dyspnea and fatigue

Dyspnea and fatigue were assessed using the modified Borg scale (0–10 points) [35] and the Fatigue Severity Scale (FSS), respectively. The latter measures fatigue severity and its influence on daily activities using a scale ranging from 1 (strongly disagree) to 7 (strongly agree) [36]. Total scores were calculated as the average of individual responses and ranged from 1 to 7; higher scores denoted greater impact of fatigue on everyday life. Overall fatigue severity was also assessed using the visual analog scale included in the FSS, which scored from 0 (worst) to 10 (normal) [37]. The DePaul Symptom Questionnaire short-form (DSQ-SF) was also used to screen for symptoms of myalgic encephalomyelitis and chronic fatigue syndrome [38]. Participants rated on a 5-point Likert scale the frequency and severity of 14 symptoms related to fatigue at rest, post-exertional fatigue, pain, and neurocognitive, autonomic/neuroendrocrine, and immune systems. The frequency and severity scores for each symptom were averaged and multiplied by 25 to create a 100-point composite score; values close to 100 represented more burden [39, 40].

#### Sit-to-stand capacity

The one-minute sit-to-stand test was used as a measure of exercise capacity by asking participants to stand up and sit on a chair without armrests as many times as possible within one minute. This test is sensitive and reliable to assess exercise capacity in patients with chronic respiratory diseases [41, 42] and correlates with the six-minute walking test in individuals with PCS [43]. Although the physical therapist encouraged all individuals to perform the test, they were also told not to overly strain themselves to avoid triggering the symptoms of post-exertional malaise. HR and SPO_2_ were measured using the digital pulse oximeter before and after the test.

#### HRQoL

HRQoL was assessed using the EuroQol-5 Dimensions-5 Levels (EQ-5D-5 L) [44, 45]. This valid and reliable tool assesses mobility, self-care, usual activities, pain/discomfort, and anxiety/depression using a 5-point scale, and total scores (EQ-5D-5 L index) were calculated by converting item scores using a value set for the Canadian population; the higher the score, the worse the HRQoL [46]. General health was assessed using a visual analog scale (EQ_VAS_) ranging from 0 (worst imaginable health state) to 100 (best imaginable health state today).

#### Participation

Participation in activities was assessed using the Canadian Occupational Performance Measure (COPM), which is a reliable, valid, and responsive survey focused on self-perceived occupational performance in the areas of self-care, productivity, and leisure [47]. Participants had to identify five individual occupational performance problems and rate the performance (1 = not able to do it all to 10 = able to do it very well) and satisfaction (1 = not satisfied at all and 10 = extremely satisfied) with their performance on a 10-point Likert scale.

#### Wearable technology

A subgroup of five participants from the PR_SD_ used one Garmin Fenix 5 wrist-worn (Garmin, Olathe, KS) and one ActiGraph wGT3X-BT triaxial accelerometer waist-worn (non-dominant hip) device for one week to explore the feasibility of collecting data throughout the day using wearable devices in individuals with PCS. Instructions were given to use both devices and their associated apps (Garmin Connect™ and Labfront) for seven days, and a wearing time of at least 10 h for 4 days was considered valid. Watch and accelerometer wear time (min/day); mean HR; number of steps per day; and time spent in sedentary behavior, light intensity physical activity, and moderate-to-vigorous physical activity (min/day) were determined from the data [48].

### Data analysis

Descriptive statistics (mean and standard deviation, median and 25 − 75% interquartile range, 95% confidence interval of median, or absolute and relative frequencies) were used to present the characteristics of the participants, and primary and secondary outcome variables. Although this was not the main objective of the study, median changes in lung function, dyspnea, fatigue, sit-to-stand capacity, HRQoL, frequency and severity of symptoms, and participation in activities between post- and pre-PR were computed and compared using Mann-Whitney test to explore potential improvements in outcomes. Moreover, the Kruskal-Wallis with Dunn’s post hoc test analyzed whether the subgroup of participants spent more time in sedentary behavior or performing light or moderate-to-vigorous physical activity. Cohen’s r (small [≤ 0.1], moderate [between 0.1 and 0.5], or large [> 0.5]) and ɛ^2^ effects sizes (small [< 0.06], moderate [between 0.06 and 0.14], and large [> 0.14]) [49, 50] were calculated for analyses related to median changes and physical activity behavior, respectively. Data were analyzed using the Statistical Package for Social Sciences, version 28 (IBM Corp., CA, USA), and a p-value < 0.05 was considered significant.

## Results

### Primary outcomes

#### Recruitment, intervention completion, dropout rate, and safety

Of the 21 individuals recruited and screened, 19 consented to participate and were randomly allocated to the PR_VC_ (9 individuals) and PR_SD_ (10 individuals) for the initial assessment; two were excluded because did not return calls after the initial contact (recruitment rate of 90%). Nineteen individuals completed the initial assessment, but two dropped out before the first virtual PR session: one from PR_SD_ due to family obligations and another from PR_VC_, who self-reported they had recovered from symptoms. The latter participant also reported that exercise intensity shown during the explanation of the program influenced the withdrawal:

“*The exercises were not challenging enough to maintain my interest; I felt my health would benefit more from more intensive and specific exercise - running for cardio and yoga for strength and flexibility.*” (Participant 101, PR_SD_).

**Fig. 1:**
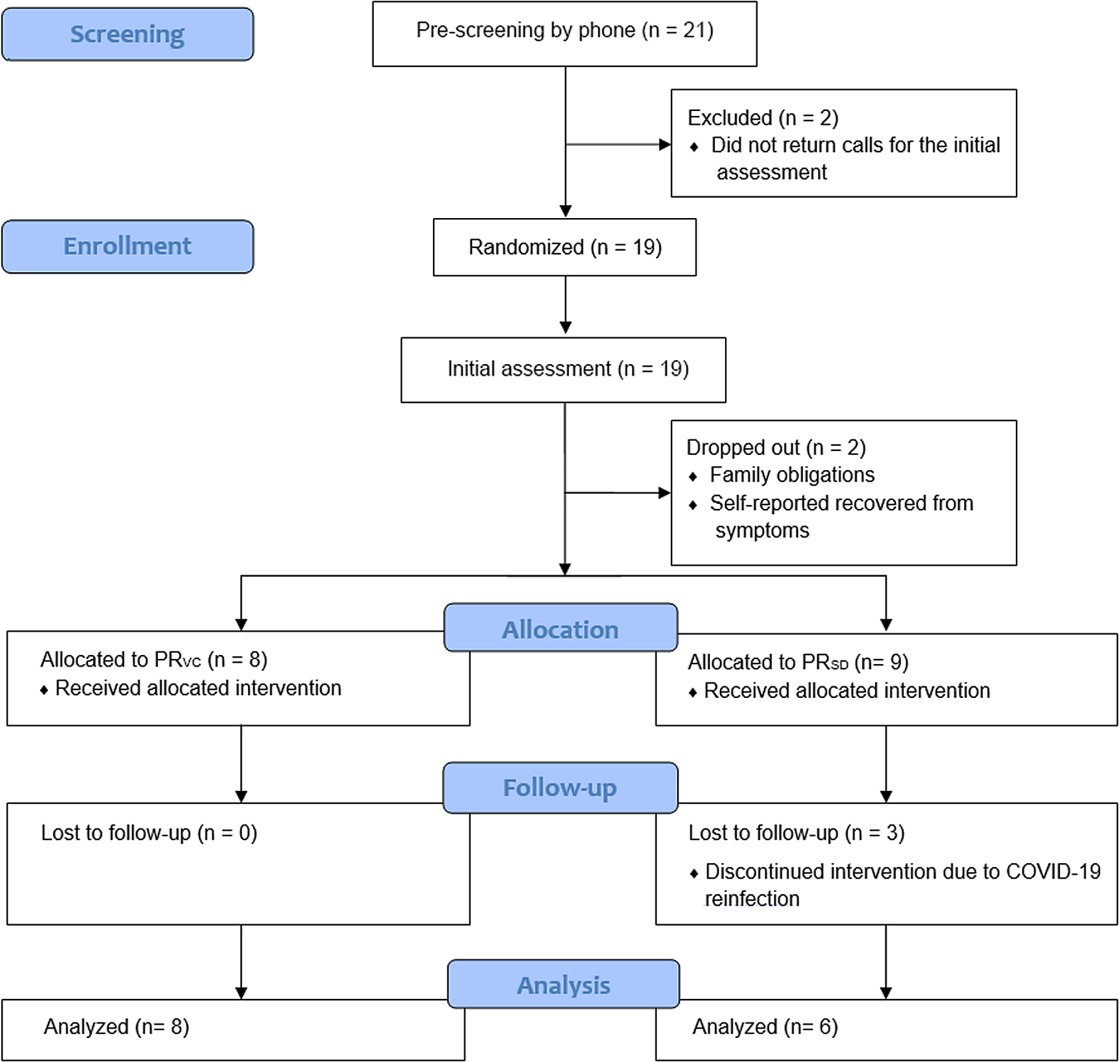
Flowchart of participant recruitment and intervention. PR_VC_: pulmonary rehabilitation via video conference; PR_SD_: self-directed pulmonary rehabilitation

Seventeen individuals initiated the virtual PR program (Fig. 1). However, three from the PR_SD_ dropped out after the second, third, and fifth sessions due to COVID-19 reinfection and did not return for the final assessment (dropout rate of 29%). The final sample comprised 14 participants: 8 in the PR_VC_ and 6 in the PR_SD_ (Fig. 1). The PR_VC_ attended 80%, while the PR_SD_ attended 84% of the total number of sessions (completion rate of the total sample was 83%); no participants reported adverse events during or after the PR program.

The baseline characteristics of the included participants are presented in Table 1. Regarding self-reported symptoms during the initial assessment, all participants from both groups complained of shortness of breath, difficulty of concentration, and fatigue at rest before and after the virtual PR. Baseline characteristics were similar between groups.

**Table 1:** Characteristics of participants

	All (*n* = 14)	PR_VC_ (*n* = 8)	PR_SD_ (*n* = 6)
Gender			
Male	2 (14)	1 (13)	1 (17)
Female	12 (86)	7 (87)	5 (83)
Age, years	49 ± 9	50 ± 9	49 ± 9
Height, centimeters	166 ± 11	166 ± 13	166 ± 9
Weight, kilograms	86 ± 28	84 ± 19	90 ± 40
Smoking			
Never	6 (43)	5 (62)	1 (16)
Current	1 (7)	-	1 (16)
Former	7 (50)	3 (37)	4 (66)
Self-reported physical activity			
Sedentary	1 (7%)	1 (12%)	-
Mild	6 (43%)	2 (25%)	4 (67%)
Moderate	3 (21%)	1 (12%)	2 (33%)
High	4 (29%)	4 (51%)	-
COVID-19 test*			
Yes	12 (86)	7 (87)	5 (83)
No	2 (14)	1 (13)	1 (17)
Active infection, days	25 ± 12.50	25.1 ± 9.61	24.4 ± 15
COVID-19 severity at baseline			
Home	13 (93)	7 (87.5)	6 (100)
Hospital	1 (7)	1 (12.5)	-
ICU	-	-	-
Use of respiratory equipment			
Yes	2 (14)	2 (25)	-
No	12 (86)	6 (75)	6 (100)
Time since infection			
3 months	1 (8)	1 (12)	-
3–6 months	5 (36)	2 (25)	3 (50)
> 6 months	8 (57)	5 (62)	3 (50)
Comorbidities			
Migraine	2 (14)	1 (13)	1 (17)
Hypertension	1 (7)	-	1 (17)
Asthma	1 (7)	1 (13)	-
Hashimoto syndrome	1 (7)	1 (13)	-
Tachycardia		1 (13)	-
Heart murmur		1 (13)	

Data shown as absolute (n) and relative frequency (%) or mean ± standard deviation. PR: pulmonary rehabilitation; ICU: intensive care unit. PR_VC_: pulmonary rehabilitation via video conference; PR_SD_: self-directed pulmonary rehabilitation. *COVID-19 diagnosis confirmed with a COVID-19 test

#### Satisfaction

Participants of both groups were satisfied with the information provided by the therapist and frequency of data submission (Table 2). Two participants highlighted the benefits of breathing exercises and pacing:

“*[…] learning to pace myself was the biggest help I got.*” (Participant 102, PR_SD_).

“*Breathing exercises have been very helpful especially.*” (Participant 107, PR_SD_).

**Table 2:** Absolute and relative frequencies of satisfaction with the pulmonary rehabilitation program

Questions	Group	Strongly agree	Agree	Neither agree nor disagree	Disagree	Strongly disagree
The information (education and links) provided by the therapist was helpful	PR_VC_	6 (75)	2 (25)	-	-	-
	PR_SD_	3 (50)	3 (50)	-	-	-
The frequency of exercise sessions were acceptable	PR_VC_	4 (50)	3 (37)	1 (13)	-	-
	PR_SD_	3 (50)	2 (33)	1 (17)	-	-
The duration of exercise sessions was acceptable	PR_VC_	4 (50)	3 (37)	-	1 (13)	-
	PR_SD_	3 (50)	2 (33)	1 (17)	-	-
The level of difficulty of exercises was suitable	PR_VC_	4 (50)	3 (37)	-	-	1 (13)
	PR_SD_	1 (16)	3 (51)	1 (16)	1 (17)	-
The time taken to collect data was acceptable (e.g., questionnaires)	PR_VC_	5 (62)	1 (13)	2 (25)	-	-
	PR_SD_	3 (50)	3 (50)	-	-	-
The frequency of data submission (once a week) was acceptable	PR_VC_	4 (50)	4 (50)	-	-	-
	PR_SD_	4 (67)	2 (33)	-	-	-
I found the apps easy to install and use on my phone or tablet	PR_VC_	6 (76)	1 (12)	1 (12)	-	-
	PR_SD_	4 (67)	2 (33)	-	-	-
I found the devices (spirometer, pulse oximeter, smart watch, and accelerometer) easy to use	PR_VC_	3 (37)	2 (25)	2 (25)	-	1 (13)
	PR_SD_	2 (33)	3 (50)	1 (17)	-	-
I had no major technical difficulties or challenges	PR_VC_	3 (38)	2 (25)	1 (12)	1 (12)	1 (12)
	PR_SD_	1 (17)	3 (55)	2 (33)	-	-
I am satisfied with the support received from the research team during the study	PR_VC_	4 (51)	1 (12)	2 (25)	-	1 (12)
	PR_SD_	4 (67)	2 (33)	-	-	-
I felt that participating in the program had a positive impact on my overall health	PR_VC_	5 (62)	2 (25)	-	1 (13)	-
	PR_SD_	3 (50)	2 (33)	-	1 (17)	-
Overall, I am satisfied with the program	PR_VC_	4 (50)	2 (25)	2 (25)	-	-
	PR_SD_	4 (67)	1 (16)	1 (16)	-	-

Data shown as absolute (n) and relative frequency (%). PR_VC_: pulmonary rehabilitation via video conference; PR_SD_: self-directed pulmonary rehabilitation

A total of 85% of all participants were satisfied with the frequency and duration of exercise sessions (88% in PR_VC_, 83% in PR_SD_). A higher percentage of participants in the PR_VC_ (88%) were satisfied with the level of difficulty of exercises compared with the PR_SD_ group (67%). However, one individual from the PR_VC_ reported the following:

“*I found that I needed to add more reps or stand […]. As well, I think I was the least sick of the group, so I felt I could do more*.” (Participant 117, PR_VC_).

Moreover, 88% of participants found the time taken to collect data acceptable (75% in PR_VC_, 100% in PR_SD_), while 94% expressed that the apps (88% in PR_VC_, 100% in PR_SD_) and 73% that the devices (63% in PR_VC_, 83% in PR_SD_) were easy to install and use (88% in PR_VC_, 100% in PR_SD_). Only one third of participants in both groups stated that they had no major technical difficulties or challenges. However, 81% were satisfied with the support received from the research team during occasion technical difficulty. Over 84% of individuals in both groups felt that the program had a positive impact on their health, and 79% were satisfied with the program (75% in PR_VC_, 83% in PR_SD_). Participants of the PR_VC_ group also reported satisfaction with the social aspects of the video conferences and added some suggestions:

“*The social aspect of the Zoom sessions was just as beneficial, if not more so, as the exercise and advice […]. Being able to speak to others who have all had normal test results, but are suffering like you, helps tremendously*.” (Participant 105, PR_VC_).

“*[…] I loved the interaction with fellow classmates and being encouraged to exercise.*” (Participant 116, PR_VC_).

“*[…] would recommend having access to virtual recorded exercise for the days one can’t make it to the live session […]*.” (Participant 116, PR_VC_)

“*[…] I much preferred the later evening times when I had more energy*.” (Participant 117, PR_VC_).

### Secondary outcomes

#### Lung function, fatigue, and sit-to-stand capacity

No significant differences in lung function and dyspnea were observed in either group pre- and post-intervention, except for changes in FEV_1_/FVC in the PR_SD_ group (*p* = 0.03, Cohen’s *r* = 0.58) (Table 3 and Additional file 2). Regarding fatigue, changes in the following domains of the DSQ-SF were significantly different between groups (Table 3 and Additional file 3): fatigue/extreme tiredness (*p* = 0.027, Cohen’s *r* = 0.58), difficulty paying attention (*p* = 0.045, Cohen’s *r* = 0.053), and feeling hot or cold for no reason (*p* = 0.024, Cohen’s *r* = 0.60). There was a slight improvement in sit-to-stand capacity post PR in the PR_VC_ group (median increase of 3 repetitions) compared with PR_SD group_ (-3.5 repetitions) (*p* = 0.012, Cohen’s *r* = 0.67).

**Table 3:** Median and 25% 75% interquartile range of changes in exercise capacity, dyspnea, fatigue, participation, lung function, and health-related quality of life, and participation

		ΔPR_VC_ (*n* = 8)	95%CI	ΔPR_SD_ (*n* = 6)	95%CI	p*	Cohen’s r
Lung function (%pred)							
FVC		0.40 [-4.17–10.80]	-13.80–39.13	-5.20 [-25.25– -0.60]	-25.70–2.10	0.171	0.37
FEV_1_		-3.40 [-5.47–5.27]	-7.00–31.20	-1.98 [-14.40–1.50]	-24.90–4.00	0.833	0.07
FEV_1_/FVC		-4.90 [-7.30– -1.15]	-7.50–7.90	0.90 [-0.10–12.81]	-2.60–21.11	0.030	0.58
PEF		-1.00 [-15.50–7.00]	-33.90–20.30	9.10 [7.10–20.30]	5.10–25.50	0.065	0.50
Dyspnea		0.00 [-1.00–0.50]	-2.00–0.50	0.00 [-0.50–0.12]	-0.75–0.50	0.947	0.01
FSS total score		-0.50 [-3.75–0.00]	-8.00–1.00	1.50 [-7.25–3.50]	-29.00–4.00	0.146	0.04
FSS_VAS_		0.00 [-1.37–1.50]	-3.00–5.00	0.00 [-0.75–1.25]	-3.00–2.00	0.736	0.12
Sit-to-stand capacity		3.00 [-0.50–6.75]	-3.00–12.00	-3.50 [-5.50– -1.00]	-7.00–2.00	0.012	0.67
EQ-5D-5 L index		0.04 [-0.07–0.11]	-0.12–0.30	-0.05 [-0.28–0.08]	-0.28–0.08	0.490	0.20
COPM-P		0.77 [-1.71–3.13]	-2.00–5.40	2.30 [1.30–3.00]	1.00–3.20	0.196	0.34
COPM-S		1.25 [-2.72–2.76]	-4.50–6.50	1.70 [-0.75–3.62]	-1.40–3.80	0.300	0.27
DSQ-SF							
Fatigue/extreme tiredeness		0.00 [-12.50–0.00]	-25.00–12.50	12.25 [0.00–12.50]	0.00–12.50	0.027	0.58
Next day soreness or fatigue		-6.25 [-21.87–0.00]	-25.00–0.00	-12.25 [-21.87–28.12]	-50.00–37.50	0.789	0.07
Tiredness after minimum exercise		-6.25 [-21.87–0.00]	-37.50–0.00	12.50 [-28.12–6.25]	-37.50–25.00	0.232	0.31
Feeling unrefreshed in the morning		-12.25 [-21.87–9.37]	-25.00–25.00	-6.25 [-18.75–18.75]	-37.50–37.50	0.691	0.10
Muscle pain or aching		12.25 [-25.00–12.50]	-25.00–37.50	6.25 [0.00–28.12]	0.00–37.50	0.548	0.16
Bloating		0.00 [-9.37–21.87]	-50.00–25.00	6.25 [-25.00–40.62]	-25.00–50.00	0.694	0.10
Problems remembering things		0.00 [-25.00–0.00]	-25.00–12.50	6.25 [0.00–15.62]	0.00–25.00	0.051	0.52
Difficulty paying attention		0.00 [-12.50–0.00]	-50.00–12.50	6.25 [0.00–31.25]	0.00–50.00	0.045	0.53
Irritable bowel problems		0.00 [-9.37–0.00]	-50.00–25.00	0.00 [-3.12–31.25]	-12.50–50.00	0.351	0.25
Feeling unsteady like you might fall		0.00 [-21.87–18.75]	-37.50–25.00	0.00 [0.00–12.50]	0.00–12.50	0.407	0.22
Cold limbs		0.00 [-9.37–31.25]	-25.00–50.00	0.00 [-15.62–21.87]	-25.00–50.00	0.791	0.07
Feeling hot or cold for no reason		0.00 [-18.75–0.00]	-37.50–0.00	6.25 [0.00–15.62]	0.00–25.00	0.024	0.60
Flue-like symptoms		0.00 [0.00–9.37]	0.00–25.00	0.00 [-3.12–31.25]	-12.50–50.00	0.940	0.02
Smells, foods, medications, or chemicals make you feel sick		0.00 [0.00–0.00]	-25.00–12.50	0.00 [0.00–18.75]	0.00–37.50	0.224	0.32

Data are shown as median and 25 − 75% interquartile range. 95%CI: 95% confidence interval of median; PR_VC_: pulmonary rehabilitation via video conference; PR_SD_: self-directed pulmonary rehabilitation. Δ represents median changes between post and pre-PR values; %pred: percentage of predicted values; FVC: Forced vital capacity; FEV_1_: Forced expiratory volume in the first second; PEF: Peak expiratory flow; FSS: Fatigue Severity Score; FSS_VAS_: FSS visual analog scale; EQ-5D-5 L: EuroQol-5 Dimensions-5 Levels; COPM-P and COPM-S: performance and satisfaction scores, respectively, in the Canadian Occupation Performance Measure; DSQ-SF: DePaul Symptom Questionnaire short-form. *p-value for between group differences

#### HRQoL and participation in activities

**Fig. 2:**
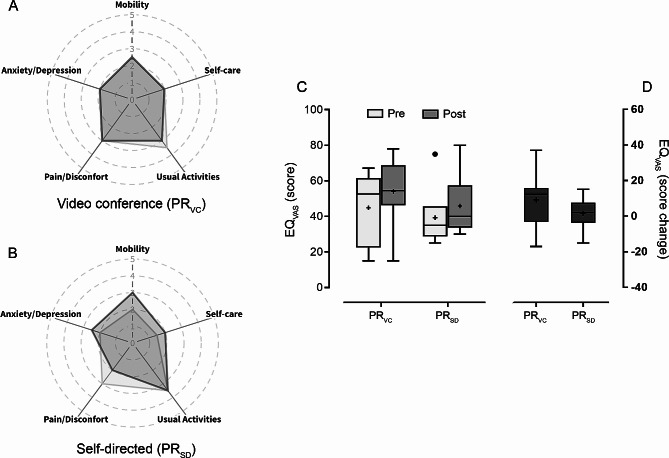
Health-related quality of life of individuals with post-COVID-19. Radar plots represented on panels A (PR_VC_) and B (PR_SD_) indicate the median scores of the domains of the EuroQol-5 Dimensions-5 Levels (the higher the score, the worse the HRQoL). Box plots on panels C and D show data median absolute and change scores of the visual analog scale of the EuroQol-5 Dimensions-5 Levels (EQ_VAS_); a score of 100 represented the best imaginable health state. The upper and lower limites of each box represents the 75th and 25th percentiles, respectively. Whiskers of box plots denote minimum and maximum values. Cohen`s r effect size for changes in EQ_VAS_ was 0.27. The plus sign (+) indicate the mean values, and the black dot (●) indicates an outlier

At baseline, the self-care, anxiety/depression, and mobility domains of the EQ-5D-5 L had lower (better) median scores in both groups, whereas the usual activities domain had higher (worse) scores. After the virtual PR, the scores of the usual activities and pain domains improved in the PR_VC_ and PR_SD_, respectively. In contrast, the mobility, self-care, and anxiety/depression domains worsened in the PR_SD_ (Fig. 2). Median EQ_VAS_ scores in the PR_VC_ and PR_SD_ increased from 52.5 to 35 before to 54.5 and 40 after the virtual PR, respectively. Although differences were not significant, median scores in the COPM performance and satisfaction with activities increased after the virtual PR in both groups (Table 3 and Additional file 2).

#### Wearable technology

Wearable devices were used by five participants of the PR_SD_ (51 ± 10 years, all females). Participants wore the watch 6 ± 1.2 days and the accelerometer 6.6 ± 0.5 days for an average time of 1,416 ± 10 and 807 ± 93 min/day, respectively. Mean HR throughout the days wearing the watch was 81 ± 10 beats/min, and participants walked an average of 4,816 ± 1,223 steps per day. They spent more time in sedentary behavior (509 ± 62 min/day) than performing moderate-to-vigorous activities (9 ± 11 min/day) (*p* < 0.001, ɛ^2^ = 0.93); no significant differences were observed with light activities (289 ± 66 min/day). No adverse events or discomfort related to wearable devices were reported.

## Discussion

Results of this pilot study suggest that it is feasible and safe to offer a fully virtual PR intervention for individuals with PCS with the exercise component delivered via video conference or pre-recorded videos. Participants were able to complete the exercises and pre- and post-assessments at home as requested and send data once a week to the team. Adherence and satisfaction with the program and use of apps and devices were high in both groups, although slightly higher in the PR_SD_ group. Except for an improvement in sit-to-stand identified in the PR_VC_ group compared with the PR_SD_, no significant changes were found in patient outcomes, which is likely explained by the small study sample size.

Conventional in- and outpatient rehabilitation is challenging for individuals with PCS, mainly because of limited access, lack of appropriate programs and resources, transportation, and conflicting schedules for working adults [51]. To overcome these barriers, video conferences and video recordings have emerged as alternative tools to provide virtual rehabilitation for this population [15, 52]. In this context, the high completion and low dropout rates, lack of adverse events, and high satisfaction observed in our virtual PR program corroborate previous findings, which reported that PR could be successfully delivered via video conference or self-directed programs [53, 54]. We believe that the social interaction during video conferences and the convenience of accessing the pre-recorded video [32, 55] motivated participants to complete an average of 20 out of 24 sessions, which is a high adherence rate for a PR program [56]. Moreover, sending the exercises and instructions may have facilitated the understanding of exercises, while the weekly call and easy access to technology may have helped buffer feelings of isolation that could negatively impact mental health, engagement, and well-being [57, 58].

The virtual delivery of PR addresses many individual and system barriers [17], enables patient flexibility, and reduces the disruption to work or daily routines [32]. Moreover, evidence indicated that virtual PR interventions were equally safe and generated similar results than in-person PR [22, 32]. Although data on PCS are still limited, the individual needs and characteristics of PR programs may influence changes in outcomes [15]. In the PR_VC_ group, the sit-to-stand capacity improved significantly, indicating that this mode of exercise delivery could help the functional recovery of individuals with PCS. This aligns with a recent systematic review that demonstrated improvements in physical performance and function of individuals with PCS after virtual PR (i.e., breathing exercises and/or general exercises) [52]. On the other hand, sit-to-stand capacity did not improve in the PR_SD_ group, probably because exercises included in the videos were conducted at low intensities. Since the video had only one level of intensity, patients were advised to adjust the exercise program (e.g., number of repetitions and amount of resistance) during the weekly call. Despite this, only 67% of participants in the PR_SD_ group were satisfied with the level of difficulty of exercises compared with 88% of participants in the PR_VC_ where the therapist was able to modify exercise intensities during sessions. Future studies should develop different videos with various levels of intensity to not only meet the progression needs of participants but also keep them interested.

The prevalence of fatigue and cognitive dysfunction, including memory and attention deficits, is high in individuals with PCS [59–61]. Although significant changes were observed in fatigue and neurocognitive domains of the DSQ-SF, these symptoms were common in the participants of both groups and occasionally challenged the implementation of the program through issues, such as patients forgetting to complete tasks or the therapist having difficulty identifying the level of exercise appropriate for each participant without risking symptom exacerbation. As these characteristics may interfere with intervention completion and dropout rates [62], virtual PR programs in PCS must screen for the frequency and severity of fatigue and cognitive dysfunction during recruitment and personalize the care according to individual needs (e.g., plan daily or weekly reminders using phone calls and mobile or tablet applications) [53, 63, 64]. In addition, since the level of technology literacy may directly impact the delivery of remote rehabilitation [65, 66], healthcare providers must first explore whether individuals with PCS have the level of knowledge and skills required for a specific PR modality.

Self-monitoring and health education can be embedded into PR programs to help promote behavioral changes and improve success rates [32, 67]. The individuals who received wearable devices in the present study were very compliant, possibly because of the simplicity of their use. This is important since wearable devices may help participants from virtual PR programs to easily monitor changes in their vital signs and understand activity behaviors [64]. For example, individuals can learn to self-adjust exercise intensity and duration based on their perceived exercise tolerance and vital signs [68]. This approach can also help healthcare providers keep participants engaged in their own care [65] and overcome barriers related to virtual interventions, such as safety concerns. In addition, improvements in symptom awareness coupled with the use of wearable devices may motivate patients to seek timely healthcare services, reducing potential complications and hospitalizations [68, 69]. Despite the promising impact of using wearable devices to optimize patient care, further studies are needed to determine reliability and continued use in this group of patients. Last, FVC results post PR of one individual from the PR_SD_ group highly affected the significance observed in FEV_1_/FVC; thus, findings should be interpreted with caution.

Some limitations should be considered when interpreting the study results. First, a small number of individuals participated in the study, which may challenge the analysis and generalization of the results, and exercise progression was performed with the PR_SD_ only during the weekly call instead of teaching the participants of this group. Although episodic disability may have affected fatigue and HRQoL results of the PR_SD_, we used non-parametric tests to reduce the chances of type I error and improve the power of the analysis. Second, a control group was not included because the main objective was to assess the feasibility and safety of virtual PR, and remote PR has already been shown to be superior to no PR [52]. Despite this, we demonstrated that virtual PR programs can be feasible, safe, and potentially beneficial for individuals with PCS. Findings may also help with the planning and implementation of long-term interventions in this population. Future studies should incorporate cost-saving analyses and explore novel ways to incorporate technology to optimize the delivery and benefits of virtual rehabilitation for PCS patients.

## Conclusions

Virtual PR with exercises delivered via video conference or pre-recorded videos are feasible and safe for individuals with PCS. Satisfaction with the various components of the programs was high in both groups, and a large percentage of participants felt that the programs had a positive impact on their health. The results also suggested that PR via video conference can improve sit-to-stand capacity in this group of patients. No other significant changes in patient outcomes were identified in either group.

### Electronic supplementary material

Below is the link to the electronic supplementary material.

Additional file 1
Additional file 2
Additional file 3

## Data Availability

No datasets were generated or analysed during the current study.

## Abbreviations

PCS: post COVID syndrome
PR: pulmonary rehabilitation
HRQoL: health-related quality of life
PR_VC_: video conference
PR_SD_: self-directed
HR: heart rate
SpO_2_: minimum oxygen saturation
FVC: forced vital capacity
FEV_1_: forced expiratory volume in the first second
FSS: Fatigue Severity Scale
DSQ-SF: DePaul Symptom Questionnaire short-form
EQ-5D-5L: EuroQol-5 Dimensions-5 Levels
COPM: Canadian Occupational Performance Measure
EQ_VAS_: visual analog scale of the EuroQol-5 Dimensions-5 Levels
