# Supplementary material for: Virtual pulmonary rehabilitation approaches in patients with post COVID syndrome: a pilot study
Source: BMC Pulm Med. 2024 Mar 18;24:139. doi: 10.1186/s12890-024-02965-3 (PMC10949685; doi:10.1186/s12890-024-02965-3)
Supplement: Supplementary file 1 — Additional file 1 Additional file 2 Additional file 3 [file 12890_2024_2965_MOESM1_ESM.docx]

**ADDITIONAL INFORMATION**

**Additional Table 1.** Pulmonary rehabilitation exercise program.

| **Exercises** | **Repetitions** | **Sets** | **Hold time** |
| --- | --- | --- | --- |
| **Warm-up** |  |  |  |
| Head turns | 10 | 1 each side | 2 seconds |
| Chin-to-chest | 10 | 1 | 2 seconds |
| Dynamic arm stretches |  |  |  |
| *Seated quadratus lumborum* | 2 | 1 each side | 5 seconds |
| *Posterior shoulder capsular* | 1 | 1 each side | 5 seconds |
| *Triceps* | 1 | 1 each side | 5 seconds |
| *Shoulder rolls* | 10 | 2 (forward and backward) | 2 seconds |
| Trunk rotation | 10 | 1 each side | 2 seconds |
| March | 10 | 2 | 2 seconds |
| Leg stretches | 2 | 1 each side | 5 seconds |
| **Resistance exercises** |  |  |  |
| Biceps curls | 10 | 2 | 2 seconds |
| Side arm raises | 5 | 2 | 2 seconds |
| Heel raises | 10 | 1 | 2 seconds |
| Knee extension | 5 | 1 each side | 5 seconds |
| Bicycle crunch | 10 | 1 | 2 seconds |
| **Aerobic exercises** |  |  |  |
| Forward arm punches | 10 | 1 | 2 seconds |
| Jumping jacks | 5 | 2 | 2 seconds |
| Sit-to-stand | 10 | 1 | 2 seconds |
| **Cool down** (stretches) |  |  |  |
| Neck stretch | 5 | 1 each side | 5 seconds |
| Posterior shoulder capsular | 2 | 1 each side | 5 seconds |
| Wrist rotation | 2 | 1 each side | 5 seconds |
| Wrist flexor extension | 2 | 1 each side | 5 seconds |
| Chest | 2 | 1 each side | 5 seconds |
| Seated quadratus lumborum | 2 | 1 each side | 5 seconds |
| Calfs | 2 | 1 each side | 5 seconds |
| Hamstrings | 2 | 1 each side | 5 seconds |

Exercises were performed with participants from the PR_VC_ and PR_SD_ seated on a chair initially. They were encouraged to use household items for upper limb exercises, and progressed from sitting to standing position when the participant and the therapist deemed it appropriate.

**Additional Table 2.** Median and 25% - 75% interquartile range of lung function, dyspnea, fatigue, STS: sit-to-stand capacity, health-related quality of life, and participation pre and post pulmonary rehabilitation (PR).

|  | **PR_VC_ (n = 8)** | |  | **PR_SD_ (n = 6)** | |
| --- | --- | --- | --- | --- | --- |
|  | **Pre PR** | **Post PR** |  | **Pre PR** | **Post PR** |
| Lung function (%pred) |  |  |  |  |  |
| FVC | 92.1 [79.6 - 98.5] | 98 [81.4 - 114] |  | 110 [97 - 126] | 97.8 [78.8 - 111] |
| FEV_1_ | 89.2 [83.2 - 100] | 89 [82.5 - 108] |  | 109 [93 - 113] | 105.5 [73.9 - 112.9] |
| FEV_1_/FVC | 100 [94.1 - 105] | 96 [89.9 - 99.4] |  | 92.4 [86 - 99.2] | 98.6 [89.8 - 109.8] |
| PEF | 96.1 [74.4 - 104] | 92.3 [73 - 100] |  | 90.6 [67 - 101] | 94 [82 – 122.5] |
| Dyspnea | 2.2 [0.6 - 2.9] | 1 [1 - 2.7] |  | 1.2 [0.4 - 3.6] | 1.5 [0 - 3.2] |
| FSS total score | 58 [52.5 - 61] | 56 [52 - 60.2] |  | 51.5 [38 - 59] | 46.5 [34.5 - 61] |
| FSS_VAS_ | 3 [1.2 - 5.6] | 3 [1.5 ­- 5.5] |  | 3 [2 - 5] | 2.5 [2 - 4.7] |
| STS capacity | 16 [9 - 21.5] | 19 [15.7 - 21] |  | 17.5 [13 - 25.5] | 14 [11 - 20] |
| HRQoL |  |  |  |  |  |
| EQ-5D-5L index | 0.618 [0.411 - 0.873] | 0.690 [0.472 - 0.844] |  | 0.692 [0.559 - 0.742] | 0.474 [0.402 - 0.825] |
| EQ_VAS_ | 52 [22.5 - 61.5] | 54 [46.2 - 68.7] |  | 35.5 [34 - 71] | 45 [33.7 - 61.2] |
| COPM-P | 3.6 [2.8 - 4.7] | 4.4 [3.4 - 6.9] |  | 2.3 [1.8 - 3.5] | 5 [3.5 - 5.7] |
| COPM-S | 3.1 [2.2 - 4.1] | 4.1 [1.1 - 5.2] |  | 1.3 [1 - 4] | 4.2 [2.4 - 7.1] |

Data are shown as median and 25% - 75% interquartile range. PR_VC_: pulmonary rehabilitation via video conference; PR_SD_: self-directed pulmonary rehabilitation. %pred: percentage of predicted values; FSS: Fatigue Severity Score; FSS_VAS_: FSS visual analog scale; STS: Sit-to-stand; EQ-5D-5L: EuroQol-5 Dimensions-5 Levels; EQ_VAS_: EuroQol visual analog scale; COPM-P and COPM-S: performance and satisfaction scores, respectively, in the Canadian Occupation Performance Measure. FVC: Forced vital capacity; FEV_1_: Forced expiratory volume in the first second; PEF: Peak expiratory flow.

**Additional Table 3.** Median and 25% - 75% interquartile range of changes of symptoms according to the DePaul Symptom Questionnaire short-form.

| **Symptoms** | **PR_VC_ (n=8)** | |  | **PR_SD_ (n=6)** | |
| --- | --- | --- | --- | --- | --- |
|  | **Pre PR** | **Post PR** |  | **Pre PR** | **Post PR** |
| **Fatigue** |  |  |  |  |  |
| 1. Fatigue/extreme tiredeness | 75.0 [65.6-84.4] | 75.0 [53.1-84.4] |  | 75.0 [46.9-87.5] | 81.2 [56.2-100]* |
| **Post-exertional fatigue** |  |  |  |  |  |
| 2. Minimum exercise makes you physically tired | 81.2 [65.6-84.4] | 75.0 [62.5-84.4] |  | 81.2 [75.0-100.0] | 75.0 [56.2-100] |
| 3. Next day soreness or fatigue after everyday activities | 75.0 [75.0-96.9] | 68.8 [50.0-75.0] |  | 56.2 [43.7-78.1] | 56.2 [46.9-78.1] |
| **Sleep** |  |  |  |  |  |
| 4. Feeling unrefreshed after you wake up in the morning | 81.2 [56.2-87.5] | 75.0 [62.5-75.0] |  | 62.5 [46.9-78.1] | 56.2 [37.5-81.2] |
| **Pain** |  |  |  |  |  |
| 5. Muscle pain or aching | 50.0 [40.6-71.9] | 62.5 [31.2-75.0] |  | 37.5 [0.0-68.7] | 50.0 [18.7-87.5] |
| 6. Bloating | 31.2 [0.0-62.5] | 25.0 [25.0-56.2] |  | 25 [0.0-25.0] | 18.7 [0.0-46.9] |
| **Neurocognitive** |  |  |  |  |  |
| 7. Difficulty paying attention for a long period of time | 75.0 [56.2-87.5] | 62.5 [50.0-84.4] |  | 62.5 [34.4-90.6] | 75.0 [43.7-100] |
| 8. Problems remembering things | 75.0 [65.6-96.9] | 75.0 [50.0-84.4] |  | 50.0 [25.0-100.0] | 87.5 [34.4-100] |
| **Autonomic/neuroendocrine** |  |  |  |  |  |
| 9. Irritable bowel problems | 43.7 [0.0-75.0] | 25.0 [6.2-71.9] |  | 12.5 [0.0-50.0] | 31.2 [0.0-56.2] |
| 10. Feeling unsteady on your feet, like you might fall | 31.2 [0.0-59.4] | 25.0 [6.2-50.0] |  | 37.5 [18.7-87.5] | 37.5 [18.7-100] |
| 11. Cold limbs | 25.0 [0.0-59.4] | 50.0 [0.0-71.9] |  | 31.2 [0.0-40.6] | 12.5 [0.0-62.5] |
| 12. Feeling hot or cold for no reason | 50.0 [25.0-62.5] | 43.8 [0.0-62.5] |  | 25.0 [0.0-56.2] | 31.2 [18.7-65.6] |
| **Immune** |  |  |  |  |  |
| 13. Flue-like symptoms | 31.2 [0.0-59.4] | 43.8 [0.0-68.8] |  | 31.2 [0.0-53.1] | 50.0 [28.1-50.0] |
| 14. Some smells, foods, medications, or chemicals make  you feel sick | 6.2 [0.0-53.1] | 0.00 [0.0-53.1] |  | 0.00 [0.0-68.7] | 18.75 [0.0-78.1] |

Data are shown as median and 25% - 75% interquartile range. PR_VC_: pulmonary rehabilitation via video conference; PR_SD_: self-directed pulmonary rehabilitation. No significant differences were observed. *p = 0.046 between pre and post PR in the PR­_SD_.
